# Removal of methylene blue by porous biochar obtained by KOH activation from bamboo biochar

**DOI:** 10.1186/s40643-023-00671-2

**Published:** 2023-08-16

**Authors:** Qing Ge, Peng Li, Miao Liu, Guo-ming Xiao, Zhu-qian Xiao, Jian-wei Mao, Xi-kun Gai

**Affiliations:** 1 Zhejiang Provincial Collaborative Innovation Center of Agricultural Biological Resources Biochemical Manufacturing, Key Laboratory of Chemical and Biological Processing Technology for Farm Products of Zhejiang Province, School of Biological and Chemical Engineering, Zhejiang University of Science and Technology, Hangzhou, 310023 Zhejiang People’s Republic of China; 2 Zhejiang Industrial Vocational and Technical College, Shaoxing, 312099 Zhejiang People’s Republic of China

**Keywords:** Bamboo biochar, Activation, Methylene blue, Adsorption, Removal performance

## Abstract

A series of activated biochar (KBBC-700, KBBC-800 and KBBC-900) which were modified by KOH and pyrolysis at various temperatures from ball-milling bamboo powder were obtained. The physicochemical properties and pore structures of activated biochar were investigated by scanning electron microscopy (SEM), fourier transform infrared spectoscopy (FT-IR), X-ray diffraction (XRD) and N_2_ adsorption/desorption. The adsorption performance for the removal of methylene blue (MB) was deeply studied. The results showed that KBBC-900 obtained at activation temperature of 900 °C exhibited a great surface area which reached 562 m^2^/g with 0.460 cm^3^/g of total pore volume. The enhancement of adsorption capacity could be ascribed to the increase of surface oxygen-containing functional groups, aromatization and mesoporous channels. The adsorption capacity was up to 67.46 mg/g under the optimum adsorption parameters with 2 g/L of adsorbent dose, 11 of initial solution pH and 298 K of the reactive temperature. The adsorption capacity was 70.63% of the first time after the material was recycled for three cycles. The kinetics indicated that the adsorption equilibrium time for MB on KBBC-900 was of about 20 min with the data fitted better to the pseudo-second-order kinetics model. The adsorption process was mainly dominated by chemical adsorption. Meanwhile, the adsorption isotherm showed that the Langmuir model fitted the best, and thermodynamic parameters revealed that the adsorption reaction was the endothermic nature and the spontaneous process. Adsorption of MB mainly attributed to electrostatic interactions, cation-π electron interaction and redox reaction. This study suggested that the activated biochar obtained by KOH activation from bamboo biochar has great potentials in the practical application to remove MB from wastewater.

**Graphical Abstract:**

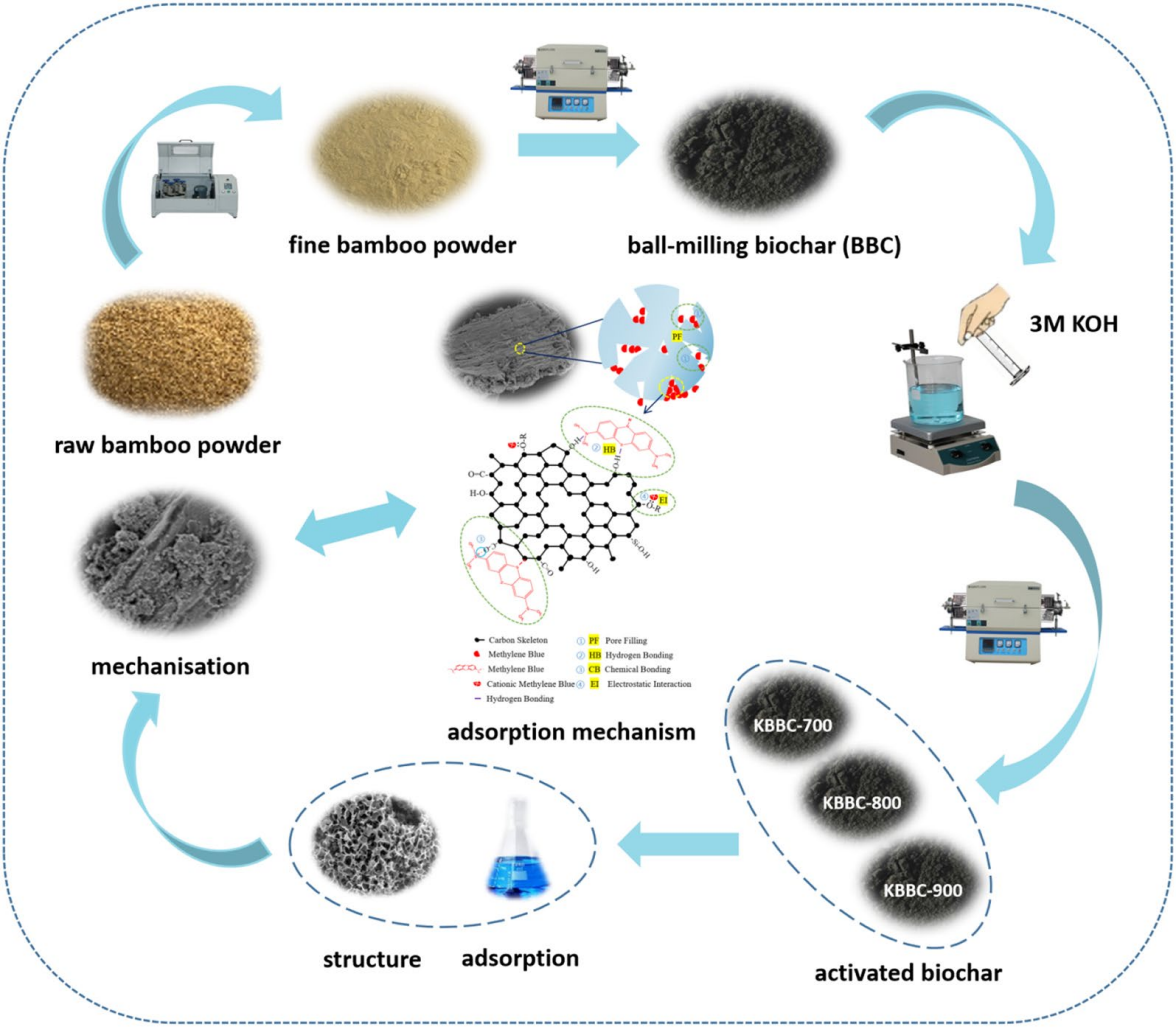

## Introduction

Water resources cover about 70% of the earth and are the key to living survival. It’s not only the basis of biological composition, but also an indispensable and valuable resource for industrial and agricultural production (Hu et al. 2021). However, water resources have been seriously destroyed in the past few decades with the development of the dye industry. The dye industrial wastewater contains various of pollutants, including azo dyes, pyridine, benzidine, heavy metal ions, cyanide, aromatic rings, landfill leachate (Yu et al. 2021; Zhang et al. 2018) and so on. Among them, the chemical structures for most of the dyes are relatively stable and complex, thus it is considerably hard to be degraded only by relying on the purification ability of the natural environment (Li et al. 2021a; Zubair and Manzar 2020; Sonu, et al. 2020).

Methylene blue (MB) is a water-soluble phenothiazine salt and widely used as chemical indicators, dyes, biological stains and drugs. It has been studied as dye pollution because of its high biotoxicity. Furthermore, when it accumulated at a certain concentration, it could cause some health problems for animals and humans, such as biological dysplasia and even cancer. At present, varieties of sewage treatment technology, including coagulation, chemical oxidation, adsorption and membrane separation have been developed to eliminate MB in the contaminated water (Qhubu et al. 2021; Liu et al. 2021a). Among them, the adsorption technology is considered to be one of the most appropriate method due to its wide applicability, cost-effective and high removal efficiency (Luo et al. 2020; Xiao et al. 2020; Zhao et al. 2021). However, due to the great difference in physicochemical properties between different adsorbents, the adsorption efficiency varies greatly. The choice of adsorption material is the key to determine the removal efficiency of methylene blue from wastewater. Therefore, it is urgent to develop adsorbents with low cost and high efficiency.

Biochar (BC) is a carbon-enriched solid obtained from the pyrolysis of biomass under the oxygen-limiting conditions without complex activation processes. It has become a popular adsorbent due to its low-cost, high surface area, abundant pore structure and so on. It has a wide range of sources, including organic waste, such as animal waste, plant roots, wheat straw, municipal sludge and other resources. There are more than 6.73 million hm^2^ of bamboo forests in China, and the area of *Phyllostachys edulis* is about 2.42 million hm^2^. In addition, *Phyllostachys edulis* is widely distributed throughout Zhejiang province. At present, the bamboo forest area in Zhejiang Province is about 0.9 million hm^2^. Among them, the area of *Phyllostachys edulis* is about 0.73 million hm^2^. In the process of bamboo processing, about 30% of the waste will be produced, resulting in a large waste of resources and terrible environment (Zhang et al. 2020a; Dam et al. 2018).

In order to response the call for national ecological civilization construction and focus on implementing the environmental governance concept of “Clear water and green mountains are equal to mountains of gold and silver”, it is necessary to further protect the natural environment and eliminate pollution without delay. Using bamboo waste to prepare biochar materials can realize the resource utilization of waste. Researchers have found that the removal efficiency of biochar was low and it had poor adsorption selectivity. Therefore, the purposeful modification of biochar was studied. At present, there are three methods have been proposed for biochar activation, including physical activation (e.g. stream or CO_2_ treatment), chemical activation (e.g. KOH, ZnCl_2_, or H_3_PO_4_) and their combination (Gao et al. 2020). Generally, the biochar activated by chemical reagents was more efficient than biochar activated by physical methods (Fu et al. 2019). Chemical activation has attracted more attention due to its short activation time, which could achieve a high surface area and abundant pore structure (Ding et al. 2023). Several studies have reported that molten alkali pyrolysis of biomass contributes to considerable surface area on biochar and KOH is frequently acted as the activating reagent in chemical activation (Wei et al. 2020; Feng et al. 2021; Zhou et al. 2021).

The main objective of this study was to obtain the activated biochar by KOH activation and investigated the adsorption characteristics and mechanism of methylene blue onto bamboo activation biochar. In the present study, three kinds of activated biochar were prepared from fine bamboo powder via KOH activation at 700, 800 and 900 ℃ (named as KBBC-700, KBBC-800 and KBBC-900), respectively. The physicochemical characteristics of the activated biochar were studied by means of SEM, FT-IR, BET, XRD and N_2_ adsorption/desorption. Meanwhile, the adsorption behaviors of KBBC-700, KBBC-800 and KBBC-900 were systematically investigated and the adsorption mechanisms of the methylene blue was explored from the perspective of kinetics and isotherms.

## Materials and methods

### Materials and reagents

The bamboo powder was obtained from Lian Yungang, Jiangsu province. Potassium hydroxide (KOH), sodium hydroxide (NaOH), hydrochloric acid (HCl) and methylene blue (MB) were of analytical grade.

### Preparation of adsorbents

Fine bamboo powder, balls milling for 2 h, was pyrolyzed at 700 ℃ for 3 h with a linear rise of 5 ℃/min under N_2_ atmosphere in a tubular furnace (SGL-1700C, Shanghai). The biochar was cooled and named as BBC. To obtain activated biochar, 5.0 g BBC was added into a 250 mL conical flask with 100 mL KOH solution (3 M). After impregnating for 12 h, the obtained biochar was washed 2 ~ 3 times using deionized water and filtrated. Then the biochar was dried at 60 ℃ for 6 h and recorded as KBBC. KBBC was pyrolyzed at three different temperatures (700, 800 and 900 ℃) for 2 h at a speed of 5 ℃/min in the N_2_-filled tubular furnace. The obtained bamboo biochar was dried at 60 ℃ for 6 h, followed by washing with deionized water and filtered until the filtrate was neutral. According to the pyrolysis tempurature, the activated biochar was named as KBBC-700, KBBC-800 and KBBC-900, respectively.

### Characterization of adsorbents

The surface morphology of the adsorbents were determined by a S-3700N scanning electron microscope (HHT, Japan). The functional groups were characterized by fourier transform infrared spectroscopy (FT-IR, Bruker V70, Germany). The pore characteristics of bamboo biochar were evaluated by a physical adsorbent apparatus (Quantachrome, USA) using N_2_ adsorption–desorption in the liquid nitrogen at 77 K. The surface area was calculated according to the BET equation. The specific surface area and pore volume of mesopore was measured according to the BJH model, while the specific surface area and pore volume of micropore was measured with t-plot method. The total pore volume and the average pore diameter was calculated by DFT model. Diffraction profiles of activated biochar were determined by X-ray diffraction (D8 ADVANCE, Bruker, Germany). The zero electric charge (pH_PZC_) of KBBC-900 was measured using pH drift method. 0.2 g KBBC-900 was added into each flask with 50 mL of NaCl (0.01 M) solution and the contents were agitated at 298 K for 24 h. The pH_PZC_ was calculated based on the difference between the initial pH and the final pH relative to the initial pH.

### Evaluation MB removal performance

#### Adsorption influencing factors

The adsorption tests were carried out in 150 mL conical flasks, containing 20 mL of MB solution (150 mg/L). The experimental parameters were selected as follows: adsorbents addition ranging from 1 to 5 g/L, pH ranging from 6 to 12, and temperature ranging from 288 to 308 K. The conical flasks were placed in a temperature-controlled shaker at a certain temperature for 240 min with a speed of 150 rpm. After the adsorption process was completed, the adsorbents were filtered with a 0.45 μm Nylon membrane and the residual concentration of MB was determined using a microplate reader (Spectra Max190, Molecular Devices, American) at 664 nm. The removal rate of MB (*R*) was calculated by Eq. (1),

1 $$ R\, = \,\frac{{C_{0} \, - \,C_{t} }}{{C_{0} }}\, \times \,100\% $$

where *R* was the removal rate (%), *C*_0_ and *C*_t_ were the initial and *t*-time concentration of MB (mg/L). The equilibrium adsorption capacity *q*_e_ (mg/g) was calculated by Eq. (2),

2 $$ q_{e} \, = \,\frac{{\left( {C_{0} \, - \,C_{e} } \right)}}{m\, \times \,1000} $$

where *C*_0_ and *C*_e_ were the initial and the equilibrium concentration of methylene blue (mg/L), *V* was the volume of MB solution (mL) and *m* was the mass of the adsorbents (g).

#### Adsorption kinetic

0.2 g adsorbents were added into a 250 mL conical flask within 100 mL of MB solution (100 mg/L) in a temperature-controlled shaker for 240 min at a speed of 150 rpm at 298 K. The appropriate amount of solution was collected at a certain time (5, 10, 15, 20, 25, 30, 40, 50, 60, 90, 120, 150, 180, 210, 240 min). After the suspended solids were separated, the concentration of MB was determined using a microplate reader (Spectra Max 190, Molecular Devices, American) at 664 nm. In this paper, the pseudo-first-order and pseudo-second-order kinetic models were used to fit the data. The calculation formulas were given by Eqs. (3) and (4), respectively.

3 $$ \ln \left( {q_{e} \, - \,q_{t} } \right)\, = \,\ln q_{e} \, - \,k_{1} t $$

4 $$ \frac{t}{{q_{t} }}\, = \,\frac{1}{{k_{2} q_{e}^{2} }}\, + \,\frac{t}{{q_{e} }} $$

where *q*_t_ and *q*_e_ represented the adsorption capacity of *t*-time and the equilibrium (mg/g), *k*_*1*_ (min^−1^) and *k*_*2*_ (g/(mg·min)) were the rate constant of the pseudo-first-order and pseudo-second-order kinetic models, respectively.

#### Adsorotion isotherm

The adsorption isotherm was used to describe the linear relationship between the adsorption capacity and the equilibrium concentration of the adsorbents at equilibrium. 0.04 g adsorbents was added into a 100 mL conical flask with 20 mL of MB solution (concentration varied from 5 to 200 mg/L) and agitated a temperature-controlled shaker at a speed of 150 rpm under three different temperatures (288 K, 298 K and 318 K) for 240 min. After the adsorbents was filtered with a 0.45 μm Nylon membrane, the absorbance of MB solution was measured at 664 nm by the microplate reader. The Langmuir and Freundlich models were utilized to investigate isotherm parameters using Eqs. (5) and (6):

5 $$ \frac{{C_{e} }}{{q_{e} }}\, = \,\frac{1}{{K_{L} q_{m} }}\, + \,\frac{{C_{e} }}{{q_{m} }} $$

6 $$ \ln q_{e} \, = \,\frac{1}{n}\ln C_{e} \, - \,\ln K_{F} $$

where *C*_e_ and *q*_e_ represented the equilibrium concentration (mg/L) of MB solution and equilibrium adsorption capacity (mg/g), respectively, *q*_*m*_ was the maximum adsorption capacity of MB (mg/g), *K*_*L*_ was the Langmuir constant (L/mg) and *K*_*F*_ was Freundlich constant (L/mg).

#### Adsorption thermodynamic

The effect of temperature on the adsorption of MB was also studied and the calculation formulas were as follows (7), (8), (9) and (10):

7 $$ K_{D} \, = \,\frac{{q_{e} }}{{C_{e} }} $$

8 $$ \Delta G_{0} \, = \, - \,RT\ln K_{D} $$

9 $$ \Delta G_{0} \, = \,\Delta H\, - \,T\Delta S $$

10 $$ \ln K_{D} \, = \,\frac{\Delta S}{R}\, - \,\frac{\Delta H}{{RT}} $$

in which *K*_*D*_ represented the adsorption equilibrium constant (L/g), *R* was the gas constant (8.314 J/(mol·K)), and *T* was the thermodynamic reactive temperature (K). *ΔG*_*0*_ was expressed the Gibbs free energy (kJ/mol), *ΔH* (kJ/mol) and *ΔS* (J/(mol·K)) represented the enthalpy change and the entropy change, respectively.

### Regeneration property

The desorption experiments were carried out with 0.1 M HCl solution. After each round of adsorption, place the adsorbent in 100 mL HCl solution (0.1 M) for 4 h. The adsorbent was cleaned with distilled water and dried at 60 ℃ for 6 h. After the adsorption, the concentration of MB in the solution was determined respectively and the removal rate and adsorption capacity were calculated.

### Data statistics and analysis

All the data were represented as means value and standard deviation and three parallel experiments per group were investigated.

## Results and discussion

### Characterization of bamboo biochar

#### SEM

**Fig. 1:**
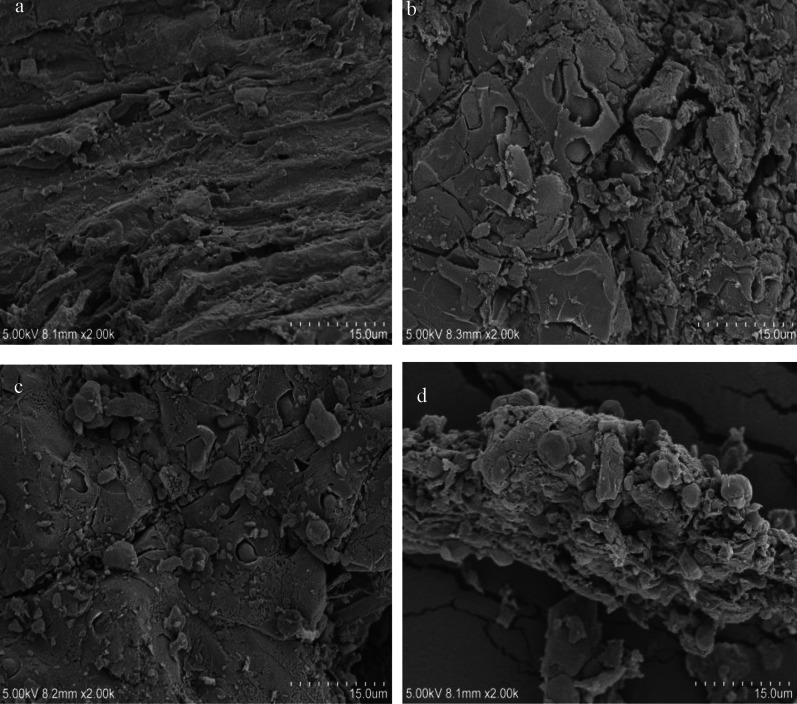
SEM images of **a** BBC, **b** KBBC-700, **c** KBBC-800, **d** KBBC-900

The SEM images of BBC, KBBC-700, KBBC-800 and KBBC-900 were shown in Fig. 1. As presented, most of the biochar obtained by the pyrolysis of bamboo powder was relatively rough. The SEM images of KBBC-700, KBBC-800 and KBBC-900 (Fig. 1b, c, d) showed that some of the biochar particles were destroyed after KOH activated. Compared with BBC sample, the biochar samples after KOH activated possessed more developed surface pore structure, which provided a larger specific surface area and more pore volume. Furthermore, with the increase of temperature, the particles on the surface were almost decomposed and showing smaller pores with rough surface. For example, the morphology of KBBC-700 in Fig. 1b showed a larger pore structure with relatively smooth surface while KBBC-800 had evident grooves with more rough surface (Fig. 1c). As for KBBC-900, the grooves on the surface was continually destroyed and showed more rough surface (Fig. 1d) due to the decomposition of organic substances in the structure, which was consistent with the result of (Li et al. 2021b). Overall, the pyrolysis temperature had a great influence on the morphology of bamboo biochar.

#### Physical structure of adsorbents

**Fig. 2:**
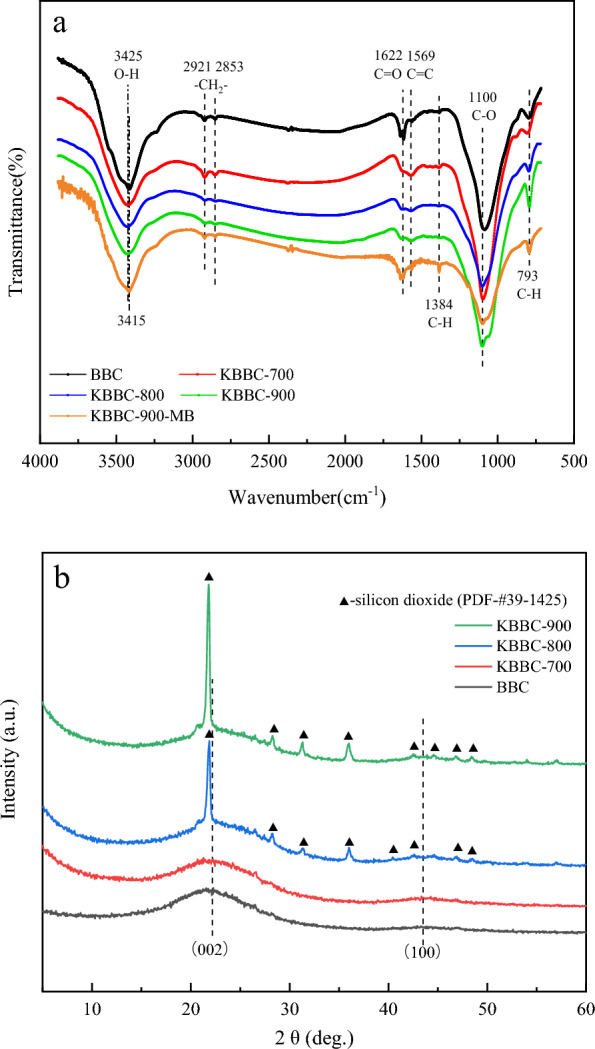
FT-IR spectra (**a**) and X-ray diffraction patterns (**b**) of adsorbents obtained from different temperatures

The FT-IR spectra of bamboo biochar and the activated biochar obtained from different temperatures were shown in Fig. 2a, and there were obvious absorption peaks change for bamboo biochar after high temperature treatment. As shown in Fig. 2a, all the samples contained a strong broad peak at 3425 cm^−1^, attributing to the stretching vibration of -OH (Dao et al. 2020; Huang et al. 2020; Miao and Li 2021). The absorption peaks observed at 2921 cm^−1^ and 2853 cm^−1^ belonged to the stretching vibrations of C-H symmetrical stretching (Mer et al. 2021), the intensity of which was weakened a little to some extent after further pyrolysis treatment at 800 ℃ and 900 °C. It was attributed to the 3D network of benzene ring transformed into 2D structure of fused ring through the cracking of functional groups which contained methyl, methylene and oxygen. Then the latter was transformed into graphite microcrystalline structure through the expansion and dehydrogenation of fused ring (Yang et al. 2016). The peak at 1622 cm^−1^ was attributed to the stretching vibration of C = O in carboxyl groups or aliphatic ketone, while the bands at 1569 cm^−1^ were related to the stretching vibrations of C = C (Liu et al. 2021b; Zhang et al. 2020b; Mallesh et al. 2022; Li et al. 2021c). The strong broad peak at 1100 cm^−1^ was attributed to stretching vibration of C–O–C glycosidic linkages (Sajjadi et al. 2021; Li et al. 2021d; Ye et al. 2020). The bands at 1000–650 cm^−1^ were regarded as γ-CH in the aromatic ring, which could provide π electron. With the increase of pyrolysis temperature, all samples showed the higher carbon content and lower heteroatom content, especially for the sample with pyrolysis temperature of 900 °C. Therefore, the intensity of all peaks in the biochar obtained under high temperature was decreased to some extent. For instance, the absorption peaks of the functional groups of C = O and C-H were gradually weakened with the temperature increased from 700 to 900 °C. However, there was still a lot of oxygen-bearing functional groups onto KBBC-900 compared with BBC. The value of the point of zero charge of KBBC-900 was 6.68, indicating that the sample was nearly neutral. It made KBBC-900 have a high adsorption capacity to cationic organic dyes. For KBBC-900-MB, the absorption peak of -OH at 3425 cm^−1^ was shifted to 3415 cm^−1^, indicating the presence of hydrogen bonding in adsorption process. Additionally, compared to KBBC-900, a new absorption peak located at 1384 cm^−1^ was observed in KBBC-900-MB which was attributed to the bending vibrating of C-H in methyl and methylene groups. It suggested that the methylene blue molecules combine with KBBC-900 through a certain force. The intensity of absorption peak at 793 cm^−1^ was significantly weakened, indicating that the π electron provided by the oxygen-containing functional groups and aromatic compounds formed a stable structure with MB. It could be concluded that the presence of more oxygen-containing functional groups and increase in the degree of aromatization played a great role in promoting the adsorption of MB molecule on the bamboo biochar (Yang et al. 2016; Wang et al. 2023).

The structural variations of all samples were also studied by X-ray diffraction patterns and the results were shown in Fig. 2b. As can be seen from Fig. 2b that all samples had two major weak bands in the range of 14–30° and 40–45°, which was corresponded to (002) and (100) reflections of typical carbon materials, indicating that all samples were amorphous structures (Wu et al. 2021). There were obvious diffraction peaks around 22.18° and 43.49° after KOH activation. With the temperature increasing from 700℃ to 900℃, the intensity of the diffraction peak increases slightly, indicating that the degree of graphitization of KBBC-800 and KBBC-900 had been improved. Besides, KBBC-800 and KBBC-900 had a strong crystal diffraction peak and the 2θ value was around 22.8°. In MDI Jade 6 software, this characteristic peak corresponded to the PDF card (39–1425).

#### Pore structure of the materials

**Fig. 3:**
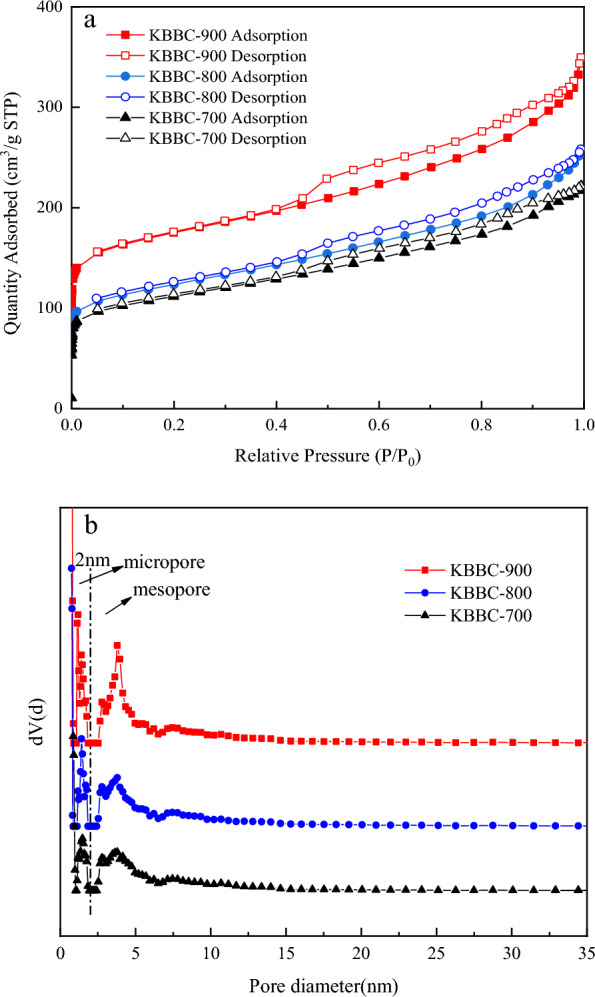
N_2_ adsorption–desorption isotherms (**a**) and pore distribution (**b**) of adsorbents obtained from different temperatures

According to the definition of the International Association for Pure and Applied Chemistry (IUPAC), pores with a pore size less than 2 nm are called micropores and pores with a pore size greater than 50 nm are called macropores, while pores with a pore size between 2 and 50 nm are called mesopores. The N_2_ adsorption/desorption isotherms and the corresponding pore size distribution of activated biochar were respectively displayed in Fig. 3a and b. As shown in Fig. 3a, all samples exhibited a mixed type I/IV adsorption behavior according to the IUPAC classification (Li et al. 2020; Buates and Imai 2020). Besides, the isotherms had the H4 hysteresis loops, which were the typical curves of the solid of the activated biochar type containing the narrow crack pores (Wu et al. 2021). The samples adsorbed large amount of nitrogen at the low pressure indicating the presence of large mesopores or micropores. As could be observed from Fig. 3b, the amount of mesopores and micropores was roughly equal and it meant that the prepared activated biochar was the micro-mesoporous material. The calculated specific surface area, pore volume and average pore size of KBBC-700, KBBC-800 and KBBC-900 were shown in Table 1. The surface area of KBBC-900 increased significantly to 562 m^2^/g when the pyrolysis temperature was 900 ℃, which was higher than that of KBBC-700 (365.6 m^2^/g) and KBBC-800 (405 m^2^/g), confirming that the porosity was developed with the increase of pyrolysis temperature. Total pore capacity also increased from 0.310 to 0.460 cm^3^/g accordingly with the temperature increased from 700 to 900 °C. Moreover, the amount of micropores and mesopores also increased significantly with the increase of pyrolysis temperature. The results showed that pyrolysis temperature had a great influence on the pore structure of the materials and proved that high temperature carbonization was a promising route for preparing biochar with well- developed porosity.

**Table 1:** The textural properties and porous parameters of samples

Sample	S_BET_ (m^2^/g)	S_meso_ (m^2^/g)	S_micro_ (m^2^/g)	V_micro_ (cm^3^/g)	V_meso_ (cm^3^/g)	Total pore volume (cm^3^/g)	Average pore diameter (nm)
KBBC-700	365.6	92.62	165.7	0.086	0.191	0.310	0.860
KBBC-800	405	117.4	179.8	0.093	0.242	0.352	0.751
KBBC-900	562	123.8	309.3	0.161	0.299	0.460	0.751

S_BET_: the total surface area of biochar

S_meso_: the mesopore surface area

S_micro_: the surface micropore area

V_micro_: the micropore volume

V_meso_:the mesopore volume

#### Zero charge point of materials

**Fig. 4:**
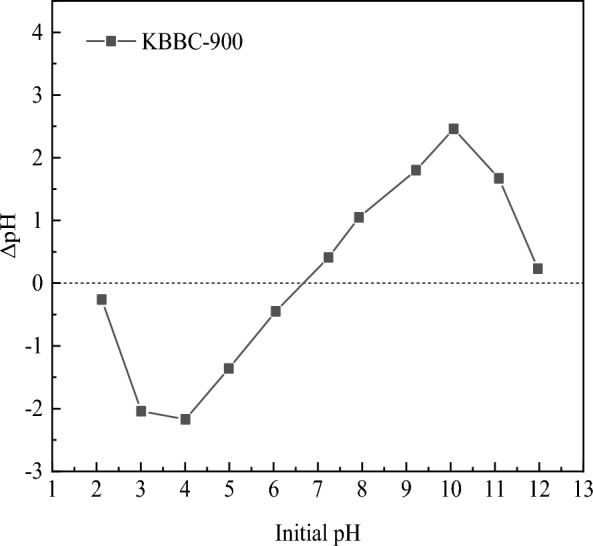
The change of ΔpH about KBBC-900 at different pH

The zero charge point referred to the pH value when the net surface charge was equal to zero, which could be used to characterize the charging condition of the biochar surface. The value of the zero charge point of KBBC-900 was shown in Fig. 4. The pH_PZC_ of KBBC-900 was 6.68, indicating that the sample was almost neutral. After KOH modification, the number of alkaline functional groups on the surface of biochar increased. Thus, when pH < 6.68, the surface of biochar was positively charged, while when pH > 6.68, the surface of biochar was negatively charged.

### Adsorption influencing factors

#### Effect of adsorbent dosage

**Fig. 5:**
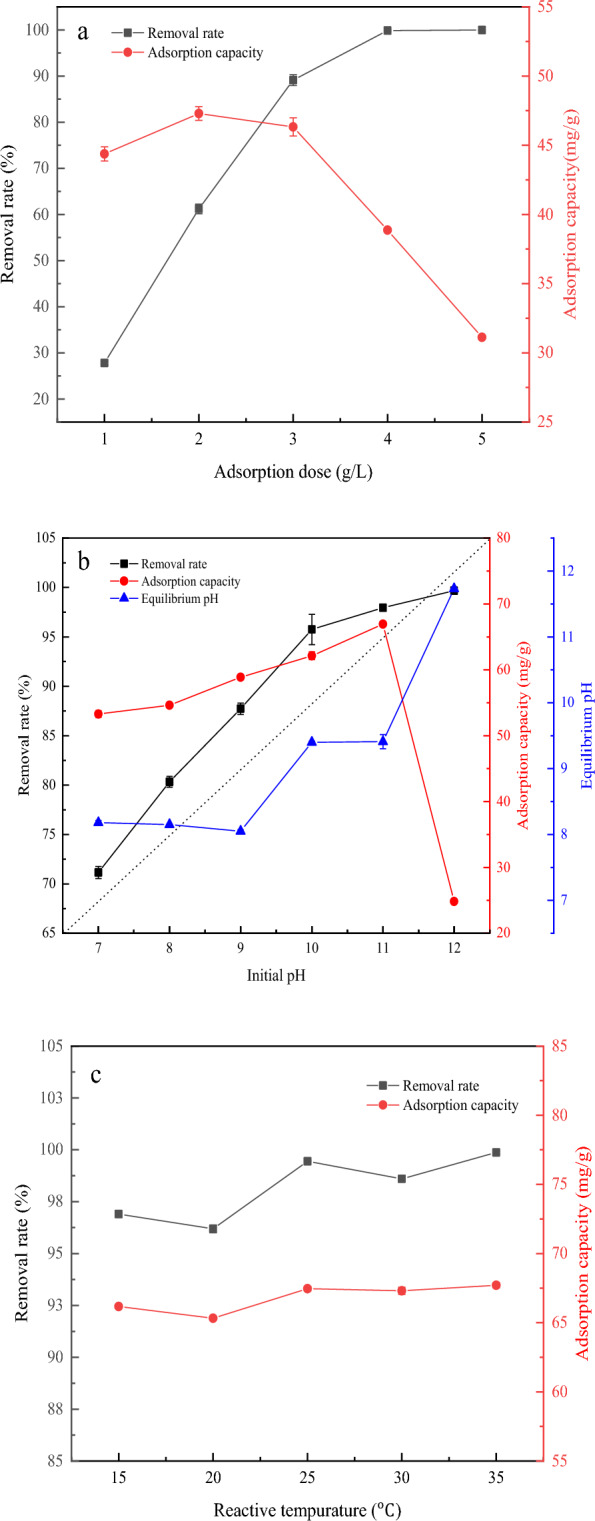
**a** Removal efficiency of KBBC-900 on MB at different adsorbent doses. 20 mL MB solution (150 mg/L), T = 298 K, contact time = 240 min. **b** Effect of initial solution pH of KBBC-900 on MB solution. 20 mL MB solution (150 mg/L), T = 298 K, adsorbent doses 2 g/L adsorbent, contact time = 240 min. **c** Efficiency of KBBC-900 on MB solution at different reactive temperatures. 20 mL MB solution (150 mg/L), pH = 11, adsorbent doses 2 g/L, contact time = 240 min

The adsorbent dosage was an important factor affecting the adsorption effect, which could affect the adsorbent-adsorbate balance in the system. The effect of adsorbent dosage on the removal rate and adsorption capacity of MB by KBBC-900 was shown in Fig. 5a. It could be observed that the removal rate increased significantly with the increase of adsorbent dose (g/L) from 1.0 to 4.0. Then the removal rate was kept at about 100% when the adsorbent dosage was increased to 4 g/L. However, the changes of adsorption capacity were different from that of the removal rate. The adsorption capacity increased first and then decreased with the adsorbent dosage ranging from 1 to 5 g/L and reached the maximum value (47.29 ± 0.49 mg/g) at the adsorbent dosage of 2.0 g/L. It was mainly due to the increase in the total number of active sites (Yu et al. 2021). However, the adsorption capacity did not continue to increase when it was up to a certain limit, which might be related to a relative decrease in the number of MB molecules per unit of adsorbent (Fan et al. 2017). Thus, the adsorbent dosage of 2 g/L was selected as the best adsorbent dosage to make full use of the maximum adsorption capacity. Under this condition, the removal rate could reach to 61.21 ± 1.05%.

#### Effect of initial solution pH

pH was an important parameter that influenced the adsorption capacity of biochar for MB, since it affected the surface charges, the ionic state of functional groups of biochar and the ionization degree of MB. The effect of initial pH of the solution on the removal rate and adsorption capacity of MB by KBBC-900 was investigated, and the result was shown in Fig. 5b. It could be observed that pH had a significant effect on MB adsorption performance of biochar. With the increased of pH from 7 to 12, the removal rate of MB increased significantly, reaching the maximum value (99.67 ± 0.01%) at pH 12. However, the adsorption capacity first increased and then decreased, reaching the maximum value (66.93 ± 0.25 mg/g) at pH 11. When the initial pH of the adsorption solution system ranges from 7.0 to 12.0, the hydroxyl and carboxyl functional groups on the surface of the biochar undergo deprotonation reaction, making its surface negatively charged. However, the cationic dye methylene blue dissociates, making the dye molecules transform into cationic MB^+^. Therefore, the higher the pH value, the stronger the electrostatic attraction between the negatively charged biochar on the surface and the cationic dye MB^+^, resulting in a greater adsorption capacity (Gurav et al. 2021). However, when the pH was too high (pH 12), the adsorbate underwent a precipitation reaction. Therefore, electrostatic effect played a crucial role in the adsorption process. The changes of the pH values before and after adsorption illustrated that the pH value of solution was closely related to the adsorption process.

#### Effect of reactive temperature

Reactive temperature was also an important factor to affect the reaction rate and equilibrium. Figure 5c showed the effect of reactive temperature on the removal rate and adsorption capacity of MB by KBBC-900 with the adsorbent dose of 2 g/L and pH 11. The removal rate of MB was all higher than 99.5% at each temperature. The adsorption capacity of KBBC-900 was increased slightly with the rise of reactive temperature and reached the maximum value (67.71 ± 0.19 mg/g), which implied that the adsorption process was endothermic (Nguyen et al. 2021). Therefore, the reaction temperature has little effect on the adsorption process, indicating that the adsorption process was mainly controlled by chemical adsorption rather than physical adsorption. Considering the practical applications, the temperature of 25 ℃ was selected due to the small variation range of adsorption capacity. Under these conditions, the adsorption capacity could reach to 67.46 ± 0.17 mg/g. Compared with the adsorption effect of other biochar on MB (Table 2) (Yu et al. 2021; Pinky et al. 2023; Astuti et al. 1203; Roy et al. 2022), we could see that KBBC-900 had a great removal effect. However, it was worth noting that it had the possibility of further improvement.

**Table 2:** Comparison of the removal effects of other adsorbents on MB dye

Biomass	Preparation method	*q*_*m*_ (mg/g)	References
*Phyllostachys edulis*	Pyrolysis at 700 ℃, 3 h and pyrolysis at 900 ℃, 2 h	67.71	This research
Corncob biomass	Pyrolysis at 700 ℃, 2 h	20.42	Pinky et al. (2023)
Petung bamboo	Microwave-assisted pyrolysis at 500 ℃, 1.5 h, 960W	45.28	Astuti et al. (2023)
*M. oleifera* seeds	–	55.10	Roy et al. (2022)
Microalgal	Microwave at 2450 MHz, 800 W	113	Yu et al. (2021)

### Adsorption kinetics

**Fig. 6:**
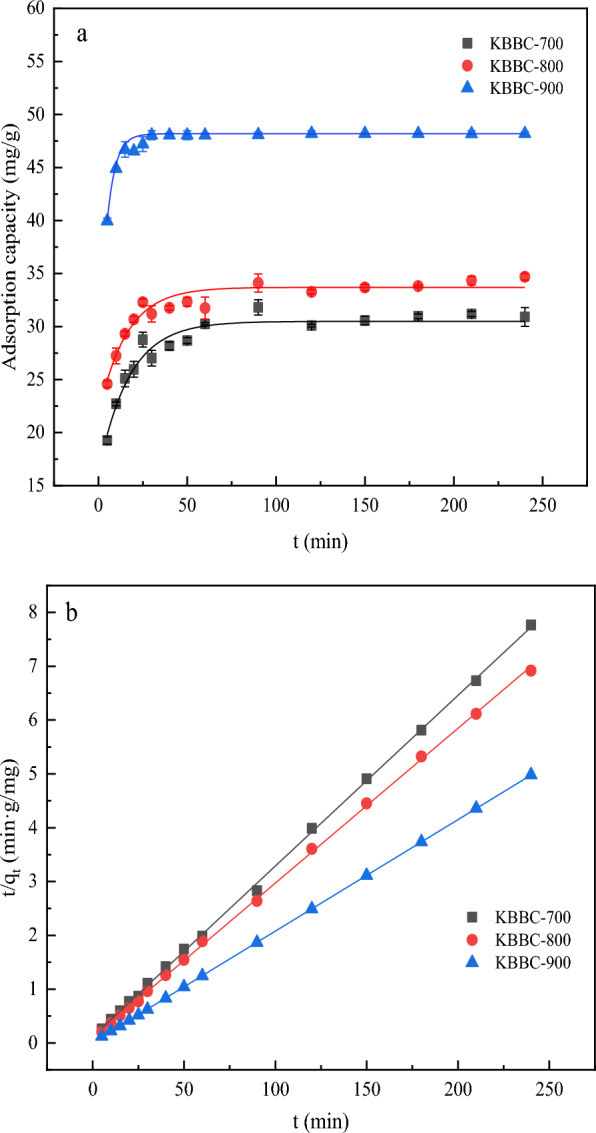
Linear regression curves of pseudo-first-order (**a**) and pseudo-second-order (**b**) for adsorption removal of MB on KBBC-700, KBBC-800, KBBC-900

The adsorption kinetic was mainly used to describe the removal of the adsorbate on the adsorbent, which was beneficial to clarify the adsorption mechanism (Xie et al. 2021). To describe the adsorption process, the adsorption capacity at different time and equilibrium state was measured. The kinetic models of pseudo-first-order and pseudo-second-order were used to fit the experimental data, and the results were showed in Fig. 6 and Table 3. As displayed, the adsorption rate of MB was relatively fast at the beginning and then, with the extension of time the adsorption capacity of MB increased slowly until the adsorption process gradually reached equilibrium. The time required for adsorption equilibrium was a critical index to evaluate the adsorption process (Li et al. 2021d; Zhang et al. 2021). The equilibrium time of KBBC-700 and KBBC-800 was about 50 min, while that of KBBC-900 was only 20 min. The fitting parameters were listed in Table 3. It can be found that pseudo-second-order model can better fit for the experimental data. Moreover, the correlation coefficient (R^2^) values of KBBC-700, KBBC-800 and KBBC-900 in pseudo-second-order model were 0.999, 0.999, 0.999, respectively, which were higher than that of pseudo-first-order model, indicating that the chemical adsorption was dominant in the adsorption process. In addition, the calculated equilibrium adsorption capacity (*q*_e_) of KBBC-700, KBBC-800 and KBBC-900 in pseudo-second-order model were 31.54 mg/g, 34.76 mg/g, 48.29 mg/g, respectively, which was more closer to the testing ones than that of pseudo-first-order model (Tomczyk et al. 2020).

**Table 3:** Fitting parameters of adsorption kinetics for MB by different activated biochar

Sample	Pseudo-first-order model			Pseudo-second-order model		
	*q*_e_ (mg/g)	K_1_ (min^−1^)	*R*^*2*^	*q*_e_ (mg/g)	K_2_ (g/(mg·min))	*R*^*2*^
KBBC-700	30.49	0.058	0.969	31.54	0.009	0.999
KBBC-800	33.70	0.064	0.940	34.76	0.009	0.999
KBBC-900	48.14	0.179	0.999	48.29	0.0069	0.999

### Adsorption isotherms

**Fig. 7:**
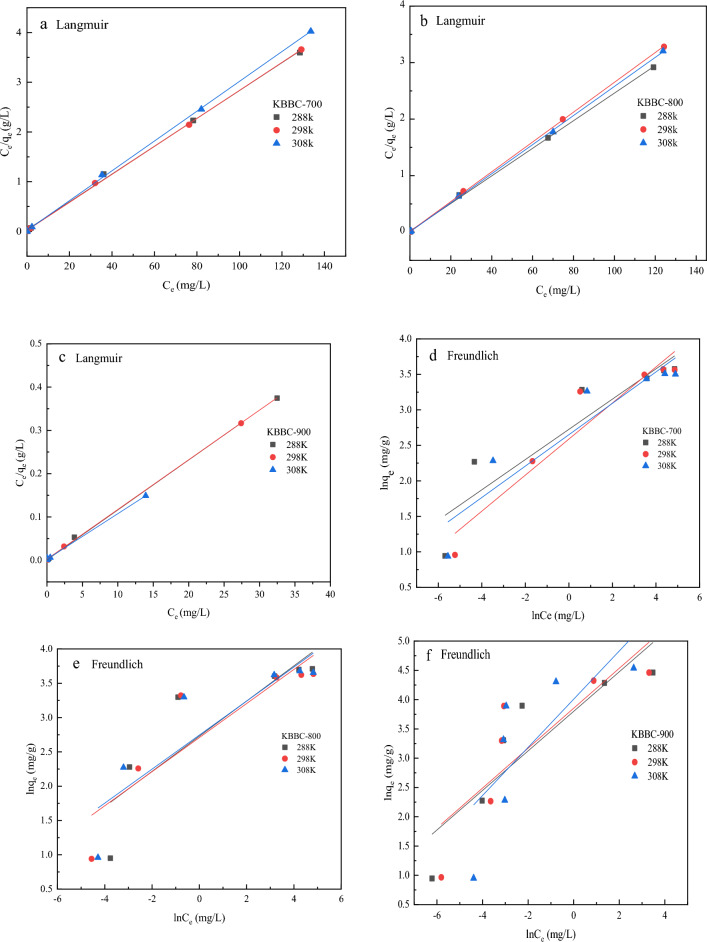
Langmuir (**a**, **b**, **c**) and Freundlich (**d**, **e**, **f**) models of MB on KBBC-700, KBBC-800 and KBBC-900

The adsorption isotherms experiments at different temperatures were conducted. Langmuir and Freundlich models were used to simulate the experimental data. The fitting parameters were listed in Table 4 and the simulated results from the Langmuir and Freundlich models were shown in Fig. 7. As displayed in Table 4, the correlation coefficient (R^2^) values of Langmuir model were higher than that of Freundlich model, which showed that the Langmuir model effectively fits the actual results and the adsorbing process was a mono-layer adsorption process. Besides, compared with the Freundlich model, the better linear fitting also meant that the Langmuir model could better describe the adsorbing process (Fig. 7). The separation factor R_L_ was an important parameter in the Langmuir model, which was used to judge whether the adsorption process was favorable. Moreover, the R_L_ values of Langmuir model at three different temperatures were all < 1, indicating the adsorption process was favorable and spontaneous.

**Table 4:** Fitting parameters of Langmuir and Freundlich models at different temperatures

Sample	T (K)	Langmuir				Freundlich		
		*q*_m_ (mg/g)	k_L_ (L/mg)	R_L_	*R*^*2*^	k_F_ (L/mg)	n	*R*^*2*^
KBBC-700	288	35.76	0.760	0.035	0.998	15.27	4.697	0.825
	298	35.51	1.416	0.022	0.999	13.31	3.938	0.892
	308	33.34	1.617	0.017	0.999	14.16	4.523	0.849
KBBC-800	288	40.97	1.282	0.031	0.999	15.31	3.901	0.700
	298	37.89	2.135	0.018	0.999	15.00	4.026	0.750
	308	38.86	5.521	0.008	0.999	15.56	4.033	0.742
KBBC-900	288	87.03	6.348	0.602	0.999	44.69	2.962	0.742
	298	95.06	4.276	0.833	0.999	47.14	2.933	0.636
	308	87.184	4.588	0.807	0.999	55.02	2.432	0.465

### Adsorption thermodynamic

The calculation of adsorption energy was a significant step in the study of adsorption performance and the thermodynamic analysis (Li et al. 2020). The calculated thermodynamic parameters of KBBC-700, KBBC-800 and KBBC-900 were listed in Table 5. The values of Δ*G*_0_ at different temperatures were all negative, which indicated that the adsorption was thermodynamically feasible and spontaneous in nature. Furthermore, the data of Δ*G*_0_ decreased with the increase of temperature, indicating that the higher temperature can produce a greater adsorption driving force. In addition, the positive values of Δ*H*_0_ demonstrated that the adsorption on MB was endothermic, which further confirmed that the higher temperature was conducive to the adsorption of MB. Moreover, the values of Δ*S*_0_ were positive and increased with the increase of the pyrolysis temperature, indicating that the randomness degree of the system at the solid–liquid interface increased when the adsorption process occurred (Shen et al. 2021).

**Table 5:** Thermodynamic parameters of KBBC-700, KBBC-800 and KBBC-900 for MB adsorption at different temperatures

Sample	T (K)	ΔG_0_ (kJ/mol)	ΔH_0_ (kJ/mol)	ΔS_0_ (J/(mol·K))	*R*^*2*^
KBBC-700	288	−6.425	3.88	35.78	0.996
	298	−6.569			
	308	−6.675			
KBBC-800	288	−9.525	6.77	56.65	0.975
	298	−9.814			
	308	−9.963			
KBBC-900	288	−14.82	16.66	109.4	0.991
	298	−15.46			
	308	−15.90			

### Regeneration property

**Fig. 8:**
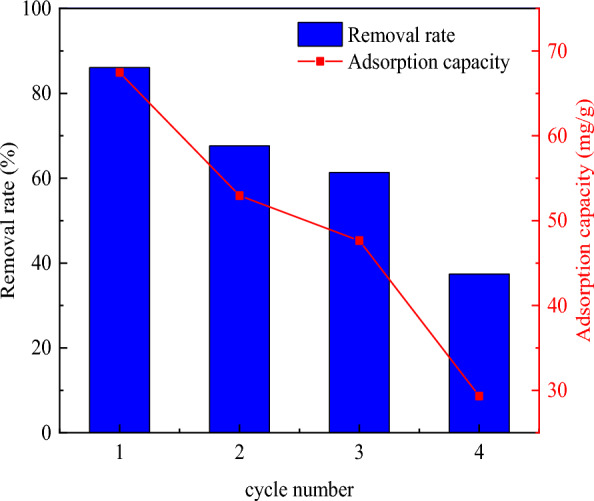
Evaluation of the stability and recyclability of KBBC-900 on MB adsorption. Reaction conditions: MB solution (150 mg/L), T = 298 K, adsorbent doses 2 g/L, contact time = 240 min

The recyclability of biochar was an important factor to investigate its application value. The regeneration property of KBBC-900 onto MB was shown in Fig. 8. It could be seen that both the adsorption capacity and desorption efficiency decreased with the increasing of usage times. After three cycles, KBBC-900 still maintained great adsorption performance with a removal rate and adsorption capacity of 61.39% and 47.65 mg/g, respectively, which were 71.31% and 70.63% of the initial values. After four cycles, the adsorption capacity of the adsorbent decreased to 29.32 mg/g. The adsorption capacity was only 43.46% of the initial value, which was due to the fact that MB was not completely desorbed at the adsorption site, resulting in the decrease of the adsorption capacity of biochar. Therefore, the optimal usage cycle of KBBC-900 was three times.

## Conclusions

In this study, the adsorbents of KBBC-700, KBBC-800, KBBC-900 were successful prepared with bamboo powder as raw materials and then activated by KOH, which had high removal capacity for MB from aqueous solution. The higher pyrolysis temperature made the bamboo biochar have well-developed pores, and KBBC-900 had the largest surface area of 562 m^2^/g with 0.460 cm^3^/g of total pore volume. The removal rate and adsorption capacity of methylene blue was affected by adsorbent dose, solution pH and reactive temperature. The best adsorption conditions were found as follows: adsorbent dose 2 g/L, solution pH 11 and reactive temperature 25 ℃. The adsorption capacity was 70.63% of the first time after the material was recycled for three cycles. The removal rate was higher than 99.5% and the maximum MB adsorption capacity was up to 67.71 ± 0.19 mg/g. It was greatly related to the aromatic graphite-type structure and the primary hydroxyl functional groups(C-O-H) onto the KBBC-900. The adsorption kinetic was well fitted to the pseudo-second-order kinetic model, indicating it was controlled by chemisorption. The adsorption isotherms showed that the Langmuir model fitted the best, suggesting the MB adsorption onto KBBC-900 followed a single-site adsorption process. Thermodynamic parameters indicated that the adsorption of MB by KBBC-900 was a spontaneous and endothermic process. In addition, the study of adsorption mechanism showed that the adsorption process was mainly chemical adsorption, accompanied by electrostatic interaction, cation-π electron interaction and redox reaction. The results indicated that the activated biochar obtained by KOH activation from bamboo biochar has great potentials in the practical application to remove MB from wastewater.

## Data Availability

All data generated or analysed during this study are included in this published article.
